# Anti-C1q antibodies: a biomarker for diagnosis and management of lupus nephritis. A narrative review

**DOI:** 10.3389/fimmu.2024.1410032

**Published:** 2024-06-13

**Authors:** Marta Calatroni, Gabriella Moroni, Emanuele Conte, Matteo Stella, Francesco Reggiani, Claudio Ponticelli

**Affiliations:** ^1^ Department of Biomedical Sciences, Humanitas University, Milan, Italy; ^2^ Nephrology and Dialysis Division, Humanitas Research Hospital, Institute for Research, Hospitalization and Health Care (IRCCS), Milan, Italy; ^3^ Department of Medicine and Surgery, University of Milano-Bicocca, Monza, Italy; ^4^ Independent researcher, Milan, Italy

**Keywords:** systemic lupus erythematous, lupus nephritis, complement system, classical complement pathway, anti-complement autoantibodies, C1q, anti-C1q antibodies, anti-C1q

## Abstract

Nephritis is a frequent and severe complication of Systemic Lupus Erythematous (SLE). The clinical course of lupus nephritis (LN) is usually characterized by alternating phases of remission and exacerbation. Flares of LN can lead to deterioration of kidney function, necessitating timely diagnosis and therapy. The presence of autoantibodies against C1q (anti-C1qAb) in the sera of SLE patients has been reported in various studies. Some research suggests that the presence and changes in the titer of anti-C1qAb may be associated with the development of LN, as well as with LN activity and renal flares. However, the exact role of anti-C1qAb in LN remains a subject of debate. Despite variability in the results of published studies, anti-C1qAb hold promise as noninvasive markers for assessing LN activity in SLE patients. Measuring anti-C1qAb levels could aid in diagnosing and managing LN during periods of both inactive disease and renal flares. Nevertheless, larger controlled trials with standardized laboratory assays are necessary to further establish the utility of anti-C1qAb in predicting the reactivation and remission of LN and guiding treatment strategies.

## Introduction

Lupus nephritis (LN) is one of the most severe complications of Systemic Lupus Erythematous (SLE), affecting a significant proportion of lupus patients, 50% of adults, and up to 60-70% of children within 5 years of diagnosis (1). The clinical presentation of LN is extremely variable, ranging from forms characterized by normal renal function with isolated urinary abnormalities or with nephrotic syndrome to others with acute renal dysfunction such as cases of nephritic syndromes or the rarer forms of rapidly progressive renal failure. Although it is intuitive that the more severe the clinical presentation, the worse the prognosis of the patient is, in many cases, there isn’t a correspondence between the severity of the clinical presentation and that of the histological lesions at kidney biopsy (2). Consequently, to assess the prognosis and decide the treatment, a kidney biopsy is mandatory at LN diagnosis. Based on the recent histological classification (3, 4), glomerular lesions can be classified into six classes, being class III and IV the most frequently diagnosed, and those associated with a worse prognosis if not adequately and timely treated. In addition to the histological class, an evaluation of the active inflammatory lesions, responsive to therapy, and the chronic irreversible lesions, the so-called activity and chronicity indexes, is recommended. LN commonly exhibits a fluctuating course characterized by periods of remission and exacerbation. There are two different types of renal flares; nephritic flares and proteinuric flares (5). Nephritic flares are defined by worsening renal function and active urinary sediment with or without an increase in proteinuria, while protenuric flares by an increase in proteinuria with stable renal function and with or without active urinary sediment. The rapid reduction of complement levels and/or a significant rise in anti-DNA antibody title can be the prodromal of a renal or extrarenal SLE reactivation, and these situations require a stricter monitoring of the patients. Reactivation of the urinary sediment with the reappearance of dysmorphic red blood cells or erythrocyte casts may be a warning of kidney reactivation, but, even in these cases, we do not recommend to increase immediately the therapy to avoid an increase in side effects, but only close observation. Despite significant improvement in renal prognosis, LN is still associated with a high rate of morbidity and mortality, leading to end-stage kidney disease (ESKD) in 5-10% of patients within 10 years of diagnosis (6, 7). The pathogenesis of LN is multifactorial although not completely understood. It involves dysregulation of the immune system, deposition of immune complexes, inflammation, and tissue damage. A main issue for the clinician involved in the management of LN is that LN course often exhibits periods of quiescence alternating with flares of activity. It is not always easy to detect the presence of a flare in patients with LN (2). Even in these cases, kidney biopsy remains the cornerstone for a correct diagnosis, prognosis and treatment of LN (8). Patients who experience multiple episodes of active nephritis, particularly those characterized by deteriorating kidney function, are at an increased risk of progressing to ESKD (9, 10). Repeating biopsy in patients with multiple flares can be difficult and poorly accepted by reluctant patients. On the other hand, rapid diagnosis and prompt treatment of renal flares are crucial in determining LN prognosis (11, 12). A noninvasive tool that could help monitor LN activity in the long term would be of utmost importance. In the last decades, there has been a growing interest in noninvasive immunological biomarkers capable of measuring disease activity, predicting flares and relapses, and influencing outcomes (13). In the realm of immunological biomarkers, anti-C1q antibodies (anti-C1qAb) have emerged as a compelling area of research interest, raising intriguing questions about their role in the pathogenesis and clinical course of LN (14–21).

In this narrative review, the intricate relationship between anti-C1qAb and SLE, with a focus on LN, will be reviewed and the potential significance of anti-C1qAb as diagnostic and prognostic biomarkers of disease activity will be outlined.

## Materials and methods

We conducted a comprehensive literature search from 1985 until December 2023, using the following terms: C1q, anti-C1q antibodies, autoimmune disease, systemic lupus erythematosus (SLE), lupus nephritis, flares, and immune biomarkers. Our search was performed in databases including PubMed, Medline, and Embase, as well as through the reference lists of retrieved articles. Additionally, we manually searched cited papers to identify additional studies relevant to the topic. The quality of the studies was assessed based on criteria including the number of participants included (more than 20) and the importance of the published findings.

## C1q and anti-C1q antibodies

**Figure 1:**
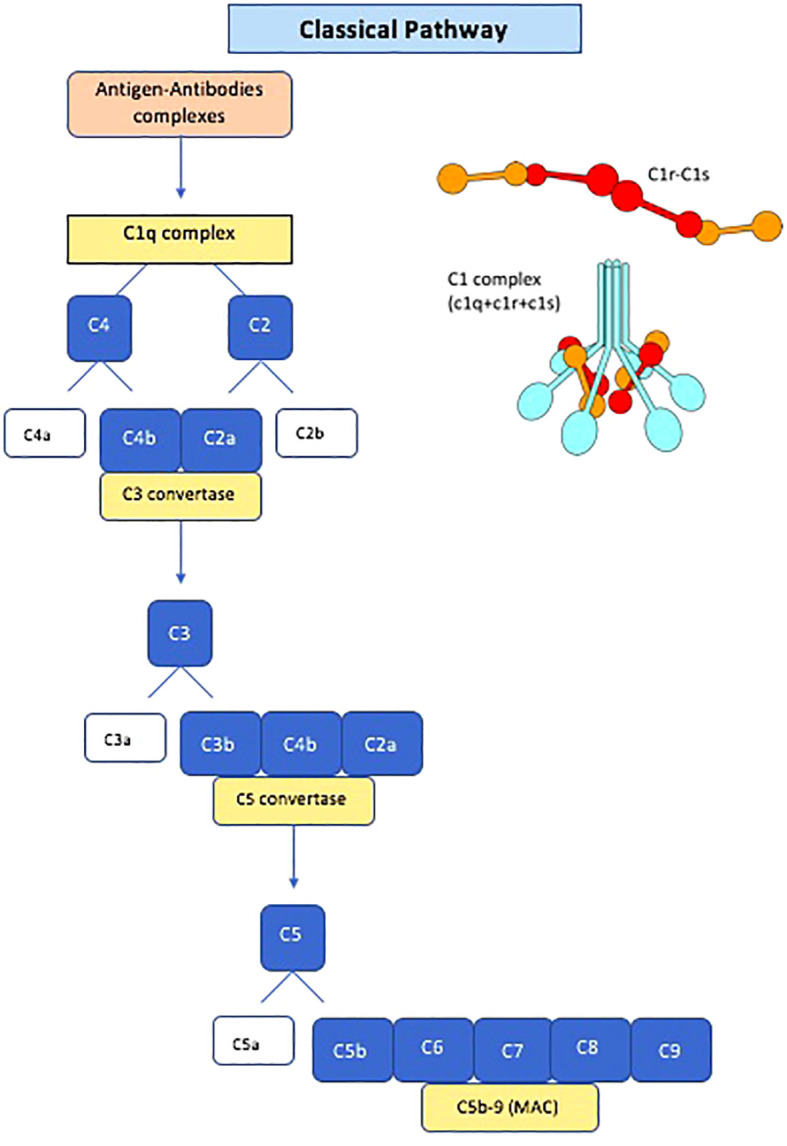
Schematic diagram of the classical pathway of the complement system and Schematic structure of C1 complex. MAC, membrane attack complex.

Anti-C1q antibodies are autoantibodies directed against C1q, the first component of the complement system. Complement activation has long been known to have a role in the pathogenesis of SLE and LN (22, 23). The classical pathway of the complement system is activated when IgM and IgG immune complexes bind to the C1 complex. The C1 complex is composed of C1q and two serine proteases, C1r and C1s (**Figure 1**). C1q is a large highly cationic glycoprotein with a molecular weight of around 410 kD and it is formed by six copies of three polypeptide chains (A, B and C) (24). After immune complex binding, the C1q conformation changes and subsequently activates C1r and C1s, with cleavage of C4 and C2, leading the formation of C3 convertase (C4b2a), then C5 convertase (C4b2a3b) and at the end the membrane attack complex with the involvement of C6-C9 (25–27) (**Figure 1**). The primary physiological role of C1q is its involvement in the clearance of immune complexes and apoptotic bodies, acting as a bridging molecule. Disruption of these processes may contribute to the development of autoimmune diseases. In the presence of impaired clearance of apoptotic cells, C1q bound to the surface of apoptotic cells may become antigenic, similar to nuclear components that are typically concealed from the immune system. Prolonged exposure of novel epitopes to the immune system may eventually lead to an autoimmune response against C1q, resulting in the formation of anti-C1q antibodies (28, 29). Notably, individuals with genetic defects in C1q are at increased risk of developing SLE, and experimental studies in mice have demonstrated an accumulation of apoptotic bodies in their kidneys (30, 31). In rodents, infusion of anti-C1qA has been associated with the formation and deposition of immune complexes in glomeruli, as well as glomerulonephritis (32). However, whether anti-C1qAb can directly activate the complement pathway in humans remains debated (33, 34). Anti-C1qAb are found in various types of infections and autoimmune diseases, including hypocomplementemic urticarial vasculitis, SLE, rheumatoid vasculitis, and Sjogren’s syndrome, but also in 3-5% of healthy individuals. High titers of anti-C1q antibodies are particularly prevalent in patients with hypocomplementemic urticarial vasculitis syndrome, reaching 100% prevalence (35–37). Anti-C1q antibodies are typically detected using an ELISA test with a high salt concentration to prevent immune complex binding (35, 38). Although several assays are available for detecting anti-C1q antibodies, none has received universal approval from the Food and Drug Administration due to a lack of systematic studies comparing their performance (39), which remains the primary obstacle to incorporating anti-C1qAb into the criteria for clinical management of SLE. Most anti-C1q antibodies belong to the IgG isotype, with a predominance of the IgG1 and IgG2 subclasses. C1q consists of an N-terminal collagen-like region (CLR) and six globular head regions (GR), with epitopes predominantly located on the CLR (14). It is suggested that anti-C1qAb primarily bind to a neoepitope in the CLR of C1q, exposed upon activation of the C1 complex and removal of C1r and C1s. Recent research indicates that anti-C1q antibodies bind specifically to solid-phase C1q rather than fluid-phase C1q, potentially leading to the exposure of cryptic epitopes that enhance Fc-receptor-mediated effector functions and contribute to autoimmune disease (34).

The correlation between antibodies against specific C1q epitopes and SLE or LN remains debated due to conflicting data (40). A large explorative study suggests that antibodies to different C1q peptide epitopes correlate with distinct disease manifestations, with antibodies against the N-terminal C1q A-chain potentially distinguishing SLE patients from healthy controls (41). Moreover, the identification of peptide A08 as a major linear epitope of C1q in SLE, along with antibodies against epitope A08 demonstrating higher sensitivity and specificity compared to conventional anti-C1qAb, has been reported (42). These findings were corroborated by Pang et al., who demonstrated that anti-A08 IgG antibodies may better differentiate LN activity (40). Consequently, a protocol for quantifying anti-C1qAb using a more sensitive assay for their detection and specific for active LN has been recently published (43).

## Anti-C1q and SLE

While hereditary C1q deficiency is uncommon among SLE patients, multiple lines of evidence underscore the significance of C1q in the inflammatory mechanisms associated with SLE. Firstly, SLE patients often display low levels of the complement pathway, including C1q. Moreover, C1q deposition is a distinctive histological finding in kidney biopsies of LN patients, and autoantibodies against C1q can be detected in the serum of patients with LN (44, 45). The presence of anti-C1q antibodies in the serum of individuals with SLE was initially identified in 1984, with a reported prevalence of 34-47% (36). In a subsequent study, anti-C1q antibodies were isolated from the glomerular basement membrane of proliferative forms of LN patients, suggesting deposition through binding to deposited C1q (46). Subsequent studies confirmed a prevalence of high anti-C1q antibodies in the serum of SLE patients ranging from 30 to 51% (16, 17, 20). Some authors reported a correlation between high levels of anti-C1q and active SLE (47). A recent Egyptian study of 70 patients with SLE confirmed that anti-C1q antibodies were present in 37% of patients with SLE and were associated with higher SLEDAI and proteinuria (48). In a large Chinese cohort of 260 SLE patients, a strong correlation with SLEDAI and anti-C1q antibodies was observed, particularly in patients with anti-A08 antibodies (40). Akhter et al., testing the sera of 47 SLE patients for the presence of anti-C1q, anti-chromatin, anti-dsDNA, anti-ribosomal P, MCP-1, VCAM, ICAM, and complement fractions, found that anti-C1q antibodies had the best association with parameters of SLE disease activity, particularly with the Physician’s Global Assessment and with modified SELENA-SLEDAI, and the highest association with proteinuria (49).

In the context of SLE, over the last two decades, studies on anti-C1q antibodies increasingly suggest that high titers of anti-C1q antibodies can predict the onset of LN. Based on these results, it was suggested that testing and monitoring anti-C1q antibody levels could be helpful as a non-invasive biomarker for kidney disease in SLE patients (28, 50).

## Anti-C1q in lupus nephritis

### Adult patients

Since the nineties, Siegert et al. (14) and Haseley et al. (51) reported significant elevations of anti-C1qAb levels in SLE patients with kidney disease compared to those without kidney involvement, particularly in the months preceding the onset of lupus nephritis. Siegert et al. evaluated anti-C1qAb titers in 68 patients with SLE and found increased titers of anti-C1qAb in 56% of patients. Ten out of the 12 patients who developed LN during the observation period had an increase in anti-C1qAb titers in the six months before LN onset, with no other new organ involvement associated with an increase in anti-C1qAb titers (14). Haseley et al., in a cohort of 240 individuals with SLE, demonstrated significantly higher levels of anti-C1qAb in patients with LN compared to those without kidney involvement (51). Subsequent studies reinforced the association between the presence and high titers of anti-C1qAb and renal disease. Trendelenburg et al. found that 97.2% of patients with LN had anti-C1qAb compared to only 25% of SLE patients without kidney involvement. Overall, anti-C1qAb showed a high sensitivity of 97.2% and a specificity of 70.3% for identifying LN (52). Sinico et al. found positive anti-C1qAb in 60% of patients with LN compared to 14% of those with SLE but without kidney disease (50).

A more comprehensive study on anti-C1qAb in LN comes from a multicenter Chinese cohort comparing 130 active LN patients with 130 non-renal SLE patients and 100 healthy controls. The predictive value of antibodies against different epitopes of C1q (intact C1q, collagen-like region, globular head region, and the new linear A08 epitope) in differentiating LN from non-LN SLE patients was explored. Although significant results were obtained with anti-intact C1qAb and anti-C1q CLR Ab, the best results in differentiating LN from non-renal SLE were achieved by anti-A08 antibodies, with a sensitivity of 73.5% and a specificity of 90.8% (40). However, some studies found a less strong association of anti-C1qAb with LN (53–55). In a Chinese cohort of 90 SLE patients, the correlation with LN was present only with very high titers of anti-C1q antibodies (55). Also, Pradhan et al. (54) found only a slightly higher prevalence of anti-C1q positivity in patients with membranoproliferative LN compared to non-LN patients.

Based on the available studies, the prevalence of anti-C1qAb in LN ranges from 56% to 97% of patients (52, 56, 57). In **Table 1**, we summarize the results of the main studies on anti-C1q in LN derived from the literature.

**Table 1:** Summaries of the most important studies, since 2000. In the top part of the table, we have listed the main studies involving SLE patients, which demonstrated a higher prevalence of anti-C1q antibodies in LN patients.

Author (year)	Country	Design of study	Population	N. of Patients	Female/Male	Median age (years)	N. of Patients with LN (n. of biopsy proven LN)	% of proliferative classes	% of positive anti-C1q in total SLE pts	% of positive anti-C1qAb in LN	Association with active renal disease	Association with renal flares	Other evidence
**Chen et al (** 16) **(2002)**	Taiwan	Retrospective	SLE Patients	47	38/9	29.15	45 (45)	–	50.8%	NA	–	–	The serum anti-C1qAb levels were higher in patients with LN with C1q deposition in the kidney biopsy.
**Marto et al (** 15) **(2005)**	UK	Retrospective	SLE patients	151	141/10	39 (15-74)	77 (77)	71.4%	49%	65%	Yes	–	Superior specificity of anti-C1qAb over anti-dsDNA for active renal disease. 30% of SLE pts with high anti-C1q developed renal disease within 9 months. No difference between proliferative and non-proliferative classes.
**Sinico et al (** 14) **(2005)**	Italy	Retrospective	SLE patients	61	NA	NA	40 (40)	85%	44%	60%	Yes	–	Patients with high anti-C1q had a higher ECLAM score.
**Mok et al (** 58) **(2010)**	China	Retrospective	SLE patients	245	233/12	40.6±12	140 (NA)	NA	21%	NA	Yes	–	Titers of anti-C1qAb and anti-dsDNA correlated significantly with the SLEDAI score. The NPV of anti-C1qAb for active renal disease was 91%. Specificity for active renal disease 84%.
**Pradhan et al (** 50) **(2012)**	India	Retrospective	SLE patients	60	55/5	25.7(14-47)	45 (45)	51.1%	58.3%	60%	No	–	Slightly higher incidence of Anti-C1qAb in LN (60vs 58%).
**Moroni et al (** 13 **) (2001)**	Italy	Retrospective	LN patients	48	44/4	37 (29-44)	48 (48)	69.0%	–	47.9%	Yes	Yes	Anti-C1qAb correlated with renal flares with a sensitivity of 87% and specificity of 93%. In all patients, anti-C1qAb titers returned to normal values after treatment-induced remission.
**Trendelemburg et al (** 48) **(2006)**	Switzerland	Prospective	LN patients	38	32/6	32,5 (19-68)	38 (38)	87%	–	97%	Yes	–	Anti-C1q strongly decreased during successful treatment.
**Moroni et al (** 59) **(2009)**	Italy	Prospective	LN patients	228	205/23	32 (15)	228 (193)	74%	–	80%	Yes	Yes	Anti-C1qAb are better than anti-dsDNA Ab, and C3 and C4 to confirm the clinical activity of LN, particularly in patients with proliferative LN and in the absence of APL.
**Fang et al (** 53) **(2009)**	China	Retrospective	LN patients	83	127/23	33.25±11	150 (150)	NA	–	56%	Yes	–	Anti-C1qAb are associated with diffuse proliferative lesions (Class IV) and with Activity index. IgG3 anti-C1q might be a more specific biomarker for monitoring disease activity.
**Cai et al (** 52) **(2010)**	China	Prospective	LN patients	73	65/8	31±14	73 (73)	61.6%	–	79.5%	Yes	–	Serum anti-C1qAb were positively correlated with the active and chronic indices in renal pathology. Patients with persistent high levels or increased titers of serum anti-C1qAb tended to develop delayed renal remission.
**Chen et al (** 57 **) (2012)**	China	Retrospective	LN patients	52	NA	29.8	52 (52)	NA	–	75%	Yes	–	Anti-C1q were higher in proliferative classes with correlation between high anti-C1qAb and activity index.
**Moroni et al (** 60) **(2015)**	Italy	Retrospective	LN patients	107	94/13	35.3±14	107 (107)	79.4%	–	70.5	Yes	–	Anti-C1qAb alone or in combination with anti-dsDNA are the most reliable test in differentiating proliferative and non-proliferative LN.
**Pang et al (** 36) **(2016)**	China	Retrospective	LN Patients	210	178/32	31.4	210 (210)	79%	–	44.3%	Yes	Yes	Intact antiC1q Ab, the C1q-collagen-like region and the A08 antibodies were higher in LN compared to SLE without LN and healthy control. A08 antibodies were better to discriminate active LN and disease relapse.
**Radanova et al (** 61) **(2022)**	Bulgaria	Retrospective	LN Patients	74	59/15	45.5±14.9	74 (74)	43%	–	27%	Yes	–	Higher levels of anti-C1qAb are associated with class IV and activity index. More severe disease (A Class BILAG Renal Score) had higher levels of Anti-C1qAb.

In the second part of the table, we have reported the studies focusing specifically on LN. dsDNA, double stranded; ECLAM, European Consensus Lupus Activity Measurement Index; SLEDAI, Systemic Lupus Erythematous Disease Activity Index; NPV, negative predictive value; LN, lupus nephritis; APL, antiphospholipid syndrome; BILAG, British Isles Lupus Assessment Group.

### Anti-C1q antibodies and active vs inactive lupus nephritis

Based on the results of the cited studies, several authors have explored the correlation between anti-C1qAb and active lupus nephritis (LN) (17–19, 50). As a biomarker for LN, anti-C1qAb also appear to be superior to classical immunological biomarkers of systemic lupus erythematosus, such as anti-dsDNA, C3, and C4, in identifying the active phase of kidney involvement (17). In 2001, Moroni et al. retrospectively compared anti-C1q titers with serum C3 and C4 levels, anti-double-stranded DNA (dsDNA), anti-endothelial cell, and antiphospholipid antibody titers in 38 samples of patients with active LN and 23 samples of patients with quiescent renal disease. Only anti-C1q correlated with active renal disease, with a sensitivity of 87% and a specificity of 92%. Furthermore, anti-C1q levels returned to normal values after treatment-induced remission in all patients (17).

In 2005, Marto et al. described 151 SLE patients, showing a higher prevalence of anti-C1qAb in patients with LN and higher titers of anti-C1qAb during active phases of renal disease, as measured with the BILAG A or B index, than during quiescent renal disease. Additionally, 9 out of 33 SLE patients with high anti-C1qAb titers developed renal disease over the next 9 months of observation, while LN did not develop in any of the 50 patients negative for anti-C1qAb. Moreover, anti-C1qAb correlated with other markers of SLE activity, though the prevalence of anti-DNA antibodies was not significantly different in patients with and without active nephritis. This result, consistent with other papers, supports the superiority of anti-C1qAb over anti-dsDNA antibodies for confirming the presence of active LN (19).

In a recent meta-analysis encompassing 25 studies, anti-C1qAb demonstrated fair sensitivity and specificity for detecting LN and distinguishing active disease from inactive LN, although renal biopsy was not performed in all patients included in these studies (61). Another meta-analysis by Eggleton et al. evaluated the diagnostic accuracy of anti-C1q in 2769 patients with SLE and suggested that anti-C1qAb, when used as a stand-alone biomarker, may have variable sensitivity and specificity values across studies. The authors concluded that while anti-C1qAb may have potential as a diagnostic test for monitoring and detecting LN in SLE patients, it would be better to consider them as part of a panel of autoantibodies (62). Regarding autoantibodies panel, the authors refer to the study of Isenberg et al, in which LN monitoring relies on anti-DNA antibodies (60). We think that in addition to anti-C1q, anti-dsDNA, anti-Smith (anti-Sm), anti-Ro/SSA, anti-La/SSB, anti-ribonucleoprotein (anti-RNP), anti-phospholipid antibodies, and complement fractions should be included in the panel of autoantibodies that any patients with suspected of LN should tested at baseline. Recently, anti-alfa enolase (ENO1), anti-histone 2 IgG2, antibodies directed against superoxide dismutase 2 (SOD2), anti-chromatin, anti-nucleosome and ribosomal P emerged in experimental studies, as possible biomarkers of active lupus nephritis, but are not yet available in the clinical setting (58, 59, 63). Considering the healthcare expenditure of testing the entire panel of available antibodies, complete screening should be performed at SLE diagnosis, while only anti-dsDNA, anti-C1q, and complement fractions should be tested to monitor LN during follow-up.

### Correlations of anti-C1q antibodies and histological parameters at kidney biopsy

Kidney biopsy remains the gold standard for the diagnosis and management of lupus nephritis. In 1997, Gunnarsson demonstrated that in patients with active LN, anti-C1q antibody titers were higher in patients with proliferative glomerulonephritis (64). Trendelemburg et al., in a prospective multicenter study, reported on 38 SLE patients with suspected active LN in whom anti-C1qAb were tested on the day of kidney biopsy. Of them, 36 patients had proliferative LN, and all but one had positive anti-C1qAb, compared to 35% of those with inactive kidney disease (52). Chen et al. (65) found anti-C1qAb in 75% of 52 patients with biopsy-proven LN. The anti-C1q titers were significantly higher in class IV than in class II and were positively associated with the glomerular deposition of C1q at immunofluorescence. A significant positive correlation was found between anti-C1q titers and the activity index, endocapillary hypercellularity, glomerular leukocyte infiltration, and karyorrhexis/fibrinoid necrosis. Conversely, a negative correlation existed with the chronicity index and, particularly, with glomerular sclerosis and interstitial fibrosis. The strongest correlations of anti-C1qAb with class IV LN and active renal disease were also confirmed in a Chinese cohort of 150 LN patients (57), in a recent study on 74 LN patients followed for 5 years (66), and in 75 Egyptian patients (67).

In a larger Italian cohort of 107 SLE patients, a panel of autoantibodies (anti-DNA, anti-C1q, anti-ribosome, and anti-nucleosome antibodies) and complement fractions were tested on the day of kidney biopsy and at 3, 6, and 12 months after the start of treatment to evaluate their correlation with the histological features at kidney biopsy. Although all the titers of the immunological tests, except those of anti-ribosome antibodies, were significantly higher in proliferative than in non-proliferative LN, at multivariate analysis, anti-C1qAb alone or in association with anti-dsDNA was the best to differentiate proliferative from non-proliferative LN. Only anti-C1qAb was correlated with the amount of proteinuria at diagnosis and with the activity index but not with the chronicity index at kidney biopsy. After 6 and 12 months of therapy, the value of anti-C1q progressively and significantly reduced, but this reduction did not predict the achievement of remission (68). Also, in the Chinese study of Pang et al., a strong correlation between anti-C1qAb, particularly anti-A08 antibodies, and class IV activity but not with chronicity index at kidney biopsy was present (40).

In contrast, in 73 LN patients described by Cai et al., of whom 79.5% had anti-C1qAb, the titer of these antibodies was positively correlated with both activity and chronicity index at kidney biopsy. At multivariate analysis, persistently high levels of serum anti-C1q at three months after the start of therapy were one of the independent predictors of failure to achieve complete renal remission. Based on their results, the authors concluded that patients with persistently high levels are at risk for progression of LN and suggest that these patients should require more intensive treatment as induction and during the course of the disease (56).

### Anti-C1q antibodies and renal flares

Few studies have evaluated serial measurements of anti-C1qAb as biomarker for predicting or confirming a renal flare. A large Italian study evaluated prospectively 228 LN patients for six years, between 2001 and 2006. The levels of anti-dsDNA, anti-C1q antibodies, C3, and C4 complement fractions were serially measured during active and quiescent LN to establish their role in confirming the clinical diagnosis of renal flares. All four tests had a negative predictive value higher than 90%, suggesting that in patients with LN, renal exacerbations seemed unlikely in the presence of normal values of all four parameters. Although all four tests were able to differentiate between renal flares and quiescent renal disease, the sensitivity and specificity were slightly higher for anti-C1qAb than for the other tests. Multivariable analysis revealed that none of the possible combinations of the four immunological tests or clinical parameters improved the predictive power of anti-C1qAb alone. Additionally, significantly more renal flares occurred with positive values of anti-C1qAb in proliferative classes than in membranous LN (69). Similar results were reported by Mok et al. in a cohort of 140 Chinese patients with LN of unknown histological classes. In comparison to anti-DNA antibodies, anti-C1qAb had similar sensitivity but better specificity in identifying phases of LN activity (70).

In the previously cited study by Pang et al., anti-C1qAb against different epitopes were tested in both active and remission phases of 40 LN patients. It was shown that titers of antibodies against the different epitopes, including anti-intact C1qAb, anti-C1qCLR, and anti-A08 antibodies, decreased significantly during remission, but negative titers were achieved only by anti-A08 antibodies. Furthermore, the occurrence of renal exacerbations within 9 months of observation was also assessed. Ten patients developed renal flares after achieving remission, and all anti-C1qAb became significantly positive at relapse, but serum anti-A08 titers were more strongly correlated with relapse than anti-intact C1qAb and anti-C1qCLRAb titers (40).

Other data come from the prospective observational study by Birmingham et al., in which they evaluated 114 SLE patients, 73 of whom had LN. In this study, anti-C1qAb were found to be less specific than anti-C3b IgG for LN. In a subgroup of 16 patients followed with serial measures of anti-C1qAb and anti-C3b IgG who developed LN flares, titers of anti-C1qAb were increased from 6 and 4 months before flare, but only in patients who were anti-C3b positive. Thus, the presence of anti-C3Ab IgG identifies patients with LN in whom anti-C1q may serve as a biomarker of renal flare (71).

## Pediatric patients

Limited studies are available about the role of anti-C1qAb in children with SLE, and the results are controversial. Ravelli et al. studied 29 patients with SLE and found anti-C1qAb in 59% of patients but without correlation with clinical manifestations, including renal involvement (72). Instead, Kozyro et al., in a large prospective study on 112 children with different histological forms of glomerular diseases, demonstrated an association between high anti-C1qAb levels and active LN. Seven out of twelve LN patients had anti-C1qAb, and six of them had active renal disease at the time of the serum sampling compared to only one of the five anti-C1q-negative children (73). A Chinese study confirmed the potential diagnostic value of anti-C1qAb also for children with LN. In this study, both C1q and anti-C1qAb were tested in 90 SLE patients, of whom 43 had active and 47 inactive SLE. C1q levels were significantly lower, and anti-C1qAb were significantly higher in SLE patients compared to healthy children and children with other rheumatic diseases. The sensitivity and specificity of anti-C1qAb to identify SLE pediatric patients were 80% and 92.1%, respectively, compared to a sensitivity and specificity of 63.3% and 94.7%, respectively, for anti-dsDNA antibodies. Anti-C1q titers were positively correlated with SLEDAI. Ninety-three percent of SLE patients who had high anti-C1qAb levels also showed kidney damage. In conclusion, reduced C1q and increased anti-C1qAb significantly correlated with LN in children and may have diagnostic value for monitoring LN in children (74). More recently, in a retrospective study including 27 SLE pediatric patients (19 with a history of LN), anti-C1qAb were tested during active and inactive SLE and compared with Farr anti-dsDNA antibody titers. Thirty-one flares (of which 18 were renal flares) were diagnosed at the start of the study or during the 55.5 months of observation. A significantly better correlation was demonstrated between anti-C1qAb and SLEDAI than with anti-dsDNA antibody. Anti-C1q antibodies were positive and at high titers during active renal flares, showing a sensitivity of 94% and a specificity of 73%, compared to the same sensitivity but a lower specificity (19%) for anti-dsDNA antibody. However, the positivity of Anti-C1q at diagnosis did not predict renal or extra-renal flares (75). In a cohort of 192 Chinese children with biopsy-proven LN, anti-C1qAb were tested among several other clinical and immunological tests to identify the predictors of the presence of glomerular microthrombi at kidney biopsy. Anti-C1qAb were present in 67.5% of patients. In multivariate analysis, anti-C1qAb, together with hemoglobin and eGFR at kidney biopsy, were identified as independent risk factors for the presence of glomerular microthrombi. Moreover, the level of anti-C1qAb was directly correlated with the activity index at kidney biopsy and with class III and IV LN (76).

### Hypocomplementemic urticarial vasculitis syndrome

Hypocomplementemic urticarial vasculitis syndrome (HUVS) is a rare form of vasculitis that affect primarily adult females, characterized by inflammation of the small blood vessels and low levels of circulating C1q. In HUVS, IgG circulating autoantibodies directed against the C1q collagen-like domain that determine the activation of the classical pathway of the complement system are present. HUVS causes recurrent episodes of urticaria due to dermal vasculitis. The incidence is reported to be around 2-20% of patients with chronic urticaria, and few cases have been reported in children. Patients with HUVS may also have systemic, multiorgan involvement, causing arthritic joint pain, lung disease, ocular inflammation, and glomerulonephritis. Kidney involvement develops in around 50% of adults, a few years after the diagnosis, presents with mild urinary abnormalities, and has generally a benign outcome (77). In children, kidney involvement seems to have a variable presentation ranging from isolated microscopic hematuria to nephrotic syndrome with possible evolution to rapidly progressive kidney failure. The histological kidney picture at light microscopy is variable too but at immunofluorescence, a full-house pattern compatible with SLE-like disease is present (78).

The association with connective tissue diseases and, with SLE, which develops in about 50% of cases, has suggested the hypothesis still under discussion whether HUVS may be a rare subset of or an unusual type of SLE (79). The results of the study of Ozçakar ZB et al. seem to confirm this hypothesis (80). They found that in a family of 3 affected children, HUVS is associated with a mutation of DNASE1L3 encoding an endonuclease that has been associated with SLE (81).

Of note, more than 40 cases of monogenic lupus caused by DNASE1L3 defects due to nine variants and with kidney involvement in more than 70% of children, have been reported in the literature (82). These results may strengthen the association between HUVS and lupus nephritis and suggest that DNASE1L3 deficiency should be considered in children with pulmonary hemorrhage, glomerulonephritis, and recurrent urticarial rash.

## Conclusions

**Figure 2:**
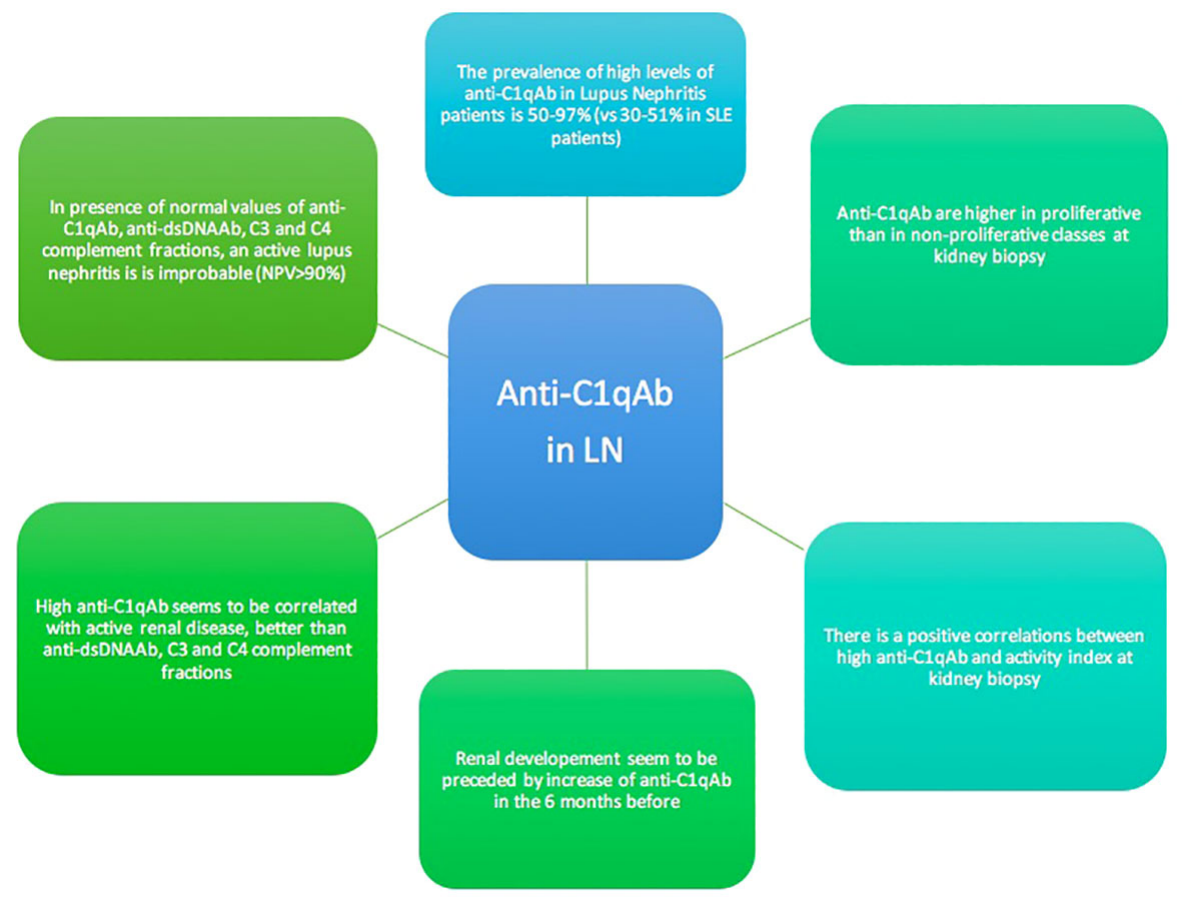
Highlights of the role of anti-C1q antibodies in Lupus nephritis patients.

A rapid diagnosis of a renal flare remains a major challenge to preserve kidney function in the long-term in LN. Although there is no universally accepted test for the determination and quantification of anti-C1qAb, the wealth of data available points towards the usefulness of monitoring these antibodies in the management of patients with LN. The presence of these antibodies in SLE seems to be strongly associated with kidney involvement in its active phases and in proliferative histological forms with high activity index. These characteristics make anti-C1qAb a useful noninvasive biomarker to be used alone or associated with the classical serum SLE biomarkers such as anti-dsDNA Ab and C3 and C4 serum fractions, in identifying LN activity. Serial anti-C1qAb measurements during follow-up are needed to establish the role of these antibodies in predicting renal flares (**Figure 2**). An effort of all the components of the scientific community (clinicians, laboratory physicians) would be desirable to develop and evaluate in clinical practice a reliable and reproducible test for the detection of anti-C1qAb. Moreover, due to the low incidence of SLE in the general population, controlled and multicenter trials are needed to establish the benefits of anti-C1qAb for prompt detection and management of lupus flares. Waiting for these data, we recommend looking for the presence of anti-C1qAb in all patients with SLE, at the diagnosis and at the appearance of signs of kidney damage, such as proteinuria, hematuria, or an increase in serum creatinine, anti-C1q should be monitored. In patients with LN, the titers of anti-C1qAb should monitored regularly during the follow-up. This may help the clinician in identifying/confirming the diagnosis of activity or remission of LN.

## Author contributions

MC: Writing – original draft, Writing – review & editing. GM: Conceptualization, Writing – original draft, Writing – review & editing. EC: Writing – review & editing. MS: Writing – review & editing. FG: Writing – review & editing. CP: Supervision, Writing – review & editing.
